# Fitting Membrane Resistance along with Action Potential Shape in Cardiac Myocytes Improves Convergence: Application of a Multi-Objective Parallel Genetic Algorithm

**DOI:** 10.1371/journal.pone.0107984

**Published:** 2014-09-24

**Authors:** Jaspreet Kaur, Anders Nygren, Edward J. Vigmond

**Affiliations:** 1 Electrical and Computer Engineering, University of Calgary, Alberta, Canada; 2 LIYRC Institute, Universite de Bordeaux 1, Bordeaux, France; Gent University, Belgium

## Abstract

Fitting parameter sets of non-linear equations in cardiac single cell ionic models to reproduce experimental behavior is a time consuming process. The standard procedure is to adjust maximum channel conductances in ionic models to reproduce action potentials (APs) recorded in isolated cells. However, vastly different sets of parameters can produce similar APs. Furthermore, even with an excellent AP match in case of single cell, tissue behaviour may be very different. We hypothesize that this uncertainty can be reduced by additionally fitting membrane resistance (R_m_). To investigate the importance of R_m_, we developed a genetic algorithm approach which incorporated R_m_ data calculated at a few points in the cycle, in addition to AP morphology. Performance was compared to a genetic algorithm using only AP morphology data. The optimal parameter sets and goodness of fit as computed by the different methods were compared. First, we fit an ionic model to itself, starting from a random parameter set. Next, we fit the AP of one ionic model to that of another. Finally, we fit an ionic model to experimentally recorded rabbit action potentials. Adding the extra objective (R_m_, at a few voltages) to the AP fit, lead to much better convergence. Typically, a smaller MSE (mean square error, defined as the average of the squared error between the target AP and AP that is to be fitted) was achieved in one fifth of the number of generations compared to using only AP data. Importantly, the variability in fit parameters was also greatly reduced, with many parameters showing an order of magnitude decrease in variability. Adding R_m_ to the objective function improves the robustness of fitting, better preserving tissue level behavior, and should be incorporated.

## Introduction

Over the past several decades, mathematical models have proven invaluable tools in the field of cardiac electrophysiology, providing significant insights into the natural processes [1], [2]. The basic modelling unit of cardiac electrophysiological simulations is the single cell ionic model which can be either phenomenological, reproducing action potentials (APs) and behavior while treating the cell as a black box, or physiological, based on explicit representations of the various ion channels, exchangers and transporters in the cells membrane and intracellular compartments. These later models have followed the pioneering work done by Hodgkin and Huxley in their model of the squid giant axon [3], consisting of a non-linear system of ordinary differential equations (ODEs).

However, determining the parameters to reproduce given AP waveforms is time consuming and ill posed. In recent years, various automated algorithms have been devised to optimise the tedious and difficult fitting. A curvilinear gradient optimization algorithm method [4] was used to fit the Beeler Reuter model [5] to a model-generated ventricular AP [6]. Syed et al. [7] developed a genetic algorithm (GA) to fit the Nygren human atrial model [8] to experimental as well as AP waveforms generated from another atrial cell ionic model [9]. A particle swarm algorithm was used to fit the Cherry et al. [10] model to model-generated atrial APs [11]. GAs have also been used to fit mouse ventricular action potentials [12].

Syed et al. [7] verified that using a more realistic pulse to stimulate the ionic model generated more accurate AP waveform fits. This idea was further enhanced by optimizing the AP from a single point in a 1D ring model of electric propagation, to take into account electrotonic interactions during excitation and propagation [13], [14]. However, the goodness of the fit was only verified by comparing the values of the fitted and original parameters, rather than the AP morphologies.

For cardiac ionic models, a particular problem is that models may perform well in single cell but fail miserably in tissue, due to the electrotonic loading. To date, researchers effectively only fit net membrane current to yield proper membrane voltage changes. Consider, though, the case that a large efflux could be counter balanced by a large influx, yielding a small net membrane current. However, a small efflux canceling a small influx could lead to the same net current. We hypothesized that fitting membrane resistance, R_m_, (defined as the reciprocal of the slope of the current-voltage relationship (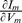)), could more properly take into account tissue coupling and moreover, produce a more robust fit. To test this hypothesis, we used a multi-objective parallel GA to fit ionic model parameters based on AP shape and R_m_. Fitting was done in both model generated and experimental data.

## Materials and Methods

### Tissue and Single Cell Simulations

The Cardiac Arrhythmias Research Package (CARP) software [15] was used for all simulations including single cell as well as tissue. For tissue simulations, a 2-dimensional grid of 1 cm×1 cm size was discretized into quadrilateral finite element mesh with edge lengths of 100 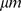. A monodomain formulation with a time discretization of 25 µs was used. A monodomain formulation is a reduction of bidomain model of electrical propagation in myocardial tissue and has reduced complexity under the assumption that the intra and extracellular domains have equal anisotropic ratios. While clearly not true, conductivity values are chosen in the monodomain equation to match bidomain anisotropic propagation. Center point stimulation was applied to tissue with intracellular conductivity in the longitudinal and transverse directions to the fibers set to 0.174 and 0.019 S/m respectively [16].

### R_m_ Evaluation

**Figure 1:**
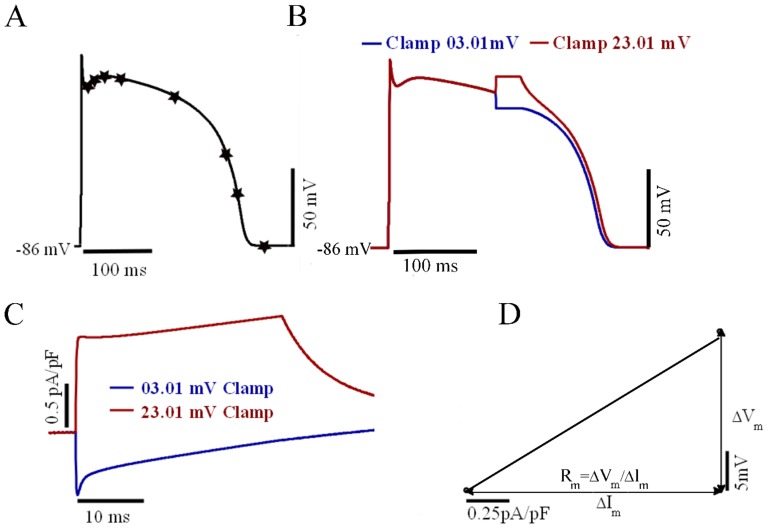
Measurement of R_m_ using voltage clamp pulses to determine the voltage-current (V-I) relationship. (A): Timing of onset of clamp pulses during different phases of the TNNP ventricle model AP are indicated by the stars. (B): Application of voltage clamp pulses 10 mV above (red curve) and below (blue curve) the membrane voltage at fifth point in the previous panel. (C): Membrane currents (Im) corresponding to clamp pulses in panel B. (D): V_m_ -I_m_ graph for the calculation of membrane resistance.

R_m_ was evaluated at several different points during an AP cycle as shown in Fig. 1A. R_m_ at a particular V_m_ was defined as the change in current in response to small perturbations in V_m_, based on the work by Zaniboni et al. [17]. In our simulation protocol (illustrated in Fig. 1B), an AP was simulated under space-clamp conditions (i.e. no spatial variation in the membrane voltage along the cell). At the instant of the V_m_ of interest, V_m_ was clamped to a value 10 mV greater. (V_m,__+10_) for 5 ms. A second simulation was then run, in which V_m_ was instead clamped 10 mV below the V_m_ of interest (V_m,__−10_). The membrane current (I_m,__+10_ or I_m,__−10_) corresponding to the clamp voltage (V_m,__+10_ or V_m,__−10_) was recorded 5 ms after the start of the clamp pulse (Fig. 1C) so that the major ionic currents had stabilized. R_m_ was calculated as the slope of the V-I graph as shown in Fig. 1D by the following equation:
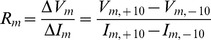
(1)


### Genetic Algorithms

A multi-objective genetic algorithm (GA) approach was used to adjust the ionic channel conductances and Ca^2+^-handling parameters in the TNNP model to obtain parameter sets that simultaneously fit a desired AP morphology and the desired values of R_m_. An initial set of parents was generated by randomly choosing values from the physiologically plausible search range for each conductance parameter. The physiological range was selected based on the available literature and the published mathematical human ventricle models [18]–[24]. By selecting a physiologically plausible range we can reach the solution faster while avoiding spurious solutions. Table 1 shows the maximum and minimum values of parameters.

**Table 1:** Maximum and Minimum value of parameters.

Parameter	Minimum Value	Maximum Value	References
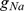 (nS/pF)	6.93	19.3	[18]–[24]
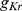 (nS/pF)	0.384	0.1728	[18]–[24]
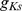 (nS/pF)	0.027	0.539	[18]–[24]
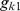 (nS/pF)	2.4	5.405	[18]–[24]
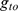 (nS/pF)	0.0196	0.303	[18]–[24]
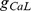 (nS/pF)	0.00008	0.00029	[18]–[20], [20]–[24]
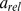 (mM/ms)	10.9	32.9	[19]–[22]
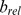 (mM)	0.17	0.51	[19]–[22]
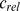 (mM/ms)	5.5	16.5	[19]–[22]

For a particular current, we calculate the chord conductance from the Equation 2, e.g., G_Na_ is given by ratio of I_Na_ and the driving force: 
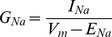
(2)where V_m_ is membrane potential and E_Na_ is Nernst equilibrium potential of sodium. The term V_m_-E_Na_ is called the electorchemical driving force. The current is often reported normalized by cell capacitance, providing the density of ionic current relative to membrane surface area (current density) in pA/pF to correct for cell size.

The population was repeatedly updated according to the principles of natural evolution: selection, crossover and mutation. The crossover rate was 0.8 and the mutation rate was chosen to be 0.01 [25]. The mutation operation was performed by adding a small random number to the parameter values, ensuring the new value remained within the physiological range. The optimization algorithm was run several times and each time the best solution obtained from the previous run was used as the initial parameter values for the next run. A multi-objective optimization was used, as there were multiple objective functions as shown in Equation 4 and 5 where optimal decisions need to be taken in the presence of trade off between the more than one objective functions. Multi-objective optimization can be described in mathematical terms as follows: 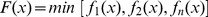
(3)


The first objective function is to minimize the normalized mean square error difference in the APs and the next objective function is to minimize the normalized absolute difference in R_m_ at *n* different V_m_ values during the AP. The ideal value for the objective function is zero, signifying that the desired criteria are fulfilled completely. Termination were based on either a maximum time limit (86400 s) or a maximum number of generations (100), whichever was reached first. For each set of parameter generated by the GA, APs were run for 3 s to reach steady-state. We also ran simulations for 10 s and observed that results were similar to those obtained after 3 s long simulations, so 3 s was chosen to reduce computation time. The multi-objective genetic algorithm finds a Pareto set of the objective functions. All solutions in a Pareto set are equally optimal, and it is up to the designer to select a solution in the Pareto set. The solution with the least value of m ean square error for AP was selected among the pareto optima [26].

To speed up the computation, the optimization algorithm was run on 8 Intel(R) Core(TM) i7- CPU 920 @ 2.67GHz cores using the Global Optimization toolbox and the Parallel Computing toolbox of MATLAB.

The performance of fitting only using AP morphology was compared with using AP morphology and R_m_. The following objective functions were minimized:

• Normalized mean square error difference in the AP,
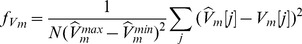
(4)where 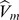 is the set of voltages defining the target AP from depolarization through return to rest, 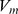 is the AP of ionic model being fit, and 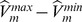 is the AP amplitude based on the control values of conductances.

• Normalized absolute difference in R_m_ at *n* different V_m_ values during the AP 
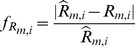
(5)where *i* = 1,2,…,*n*. The absolute difference between the target (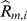) and fit R_m_ at each voltage point was normalized by R_m_ evaluated at that voltage point in the base model. For every iteration of the algorithm, the GA maintained a population of 100 potential solutions. It may be argued that a small number of potential solutions could guide the algorithm to poor solutions and that a large number of potential solutions could make the algorithm expend more computation time in finding a solution. Due to significant influence of population size on the solution quality and search time, we studied the effect of this GA parameter. Test cases for 50, 100 and 200 potential solutions were compared and results are shown in Table 2, which demonstrates that the optimal number of potential solutions found was 100. In this case, accurate model parameters values are obtained in reasonable computational time. Increasing the number of potential solutions from 50 to 100 significantly improves the resulting value of the objective functions. The MSE voltage and R_m_ were reduced by 19.8% and 21.21% respectively, whereas increasing the number of potential solutions to 200 decreased the MSE voltage and R_m_ accuracy by 1.06% and 2.14% respectively and led to significant increases in computational time. So increasing the number of potential solutions resulted in more computation cost without considerably improving the value of the objective functions.

**Table 2:** Dependence of the Number of potential solutions on accuracy.

Number of potential solutions	MSE Voltage (mV)	MSE R_m_ (G 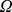 )	Computation time(ms)
50	0.013	0.201	32478
100	0.102	0.16	68946
200	0.01	0.15	154789

### Model-to-model fitting

The single cell Ten Tusscher mathematical model (TNNP) of the human ventriclular action potential [22] was used as the base model for fitting. The change in membrane voltage (V_m_) per unit time is governed by the following differential equation:
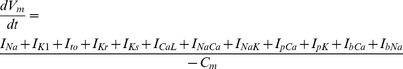
(6)


**Figure 2:**
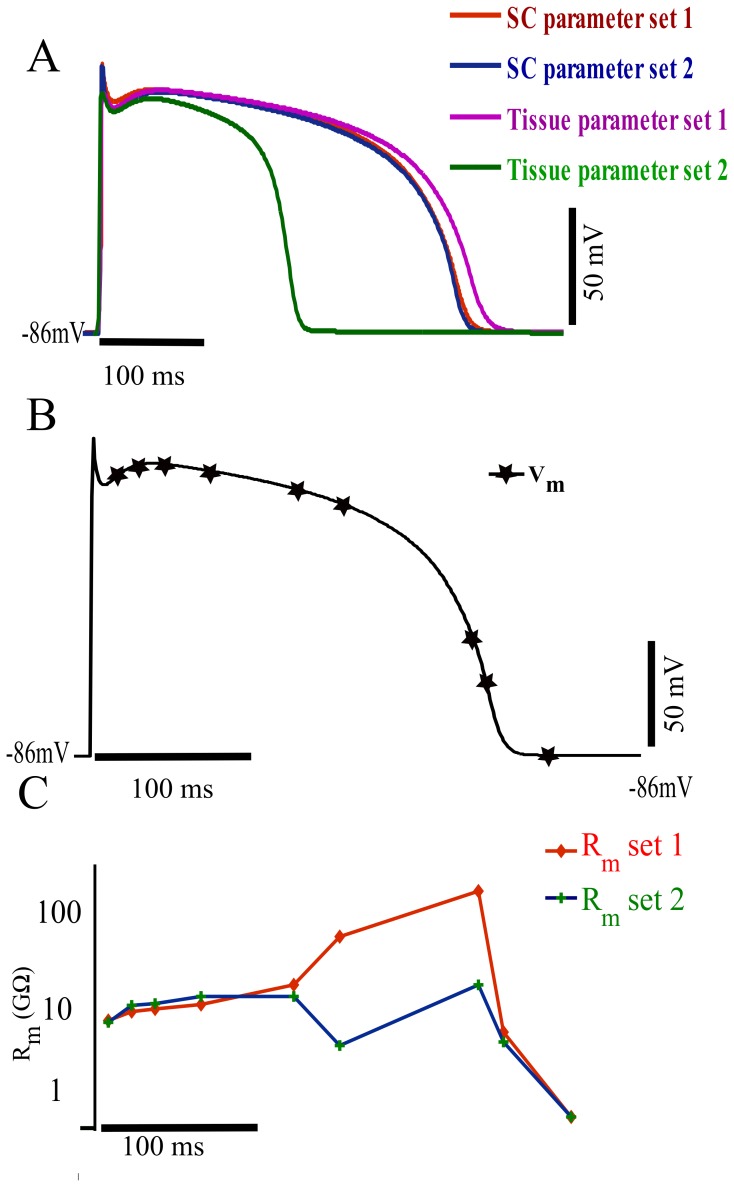
AP and R_m_ for two sets of parameters. (A): APs produced by two sets of parameters (shown in Table 3) for the TNNP model in single cell (SC) and tissue (Tissue) simulations. (B) Stars indicate voltage clamp control points as defined in Fig.A. The AP is shown in black curve. (C) R_m_ calculated at nine points during the AP for parameter set 1 and 2. Red and blue curves show membrane resistances calculated for parameter sets 1 and 2 respectively. The Y axis is logarithmic.

Two different sets of TNNP parameters Table 3 published by Sarkar & Sobie [27] were used to generate similar APs (Fig. 2A) with different underlying ionic current densities. The input parameter sets consisted of the following: 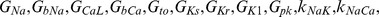 and *k_pCa_*, the maximum conductances (*G*) for sodium, background sodium, L-type calcium, background calcium, transient outward, slow-delayed rectifier, rapid-delay rectifier, inward rectifier, plateau potassium currents respectively, the maximum turnover rates (*k*) for the sodium-potassium (*NaK*) and sodium-calcium (*NaCa*) exchangers and calcium pump (*pCa*), and 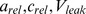, and *V^up^* (all related to intracellular Ca^2+^ handling, for details, see [22]).

**Table 3:** Two parameter sets for the TNNP ventricle model showing similar AP [3].

Parameter	Units	Control Value	Parameter Set#1		Parameter Set#2	
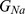	nS/pF	14.838	−11.90		18.47	
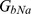	nS/pF	0.00029	−3		0.00	
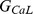	nS/pF	0.000175	0.00		−4.50	
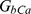	nS/pF	0.000592	7.15		−17.74	
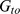	nS/pF	0.294	−25		5.90	
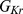	nS/pF	0.096	31.80		−26.70	
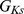	nS/pF	0.245	28.80		14.80	
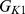	nS/pF	5.405	−2.28		42.89	
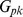	nS/pF	0.0146	7.10		−14.90	
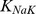	pA/pF	1.362	3.50		21.60	
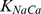	pA/pF	1000	−6.68		30.30	
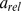	mM/ms	0.016464	21.62		−30.00	
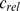	mM/ms	0.008232	−11.90		−28.40	
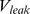	ms	0.00008	58.00		12.20	
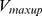	mM/ms	0.000425	−6.68		−10.90	
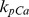	pA/pF	0.825	1.16		16.14	

Control value in the second column shows the value as published. Parameter sets #1 and #2 show percentage change from the control value.

The fitting technique was first verified by starting with random parameters values for the TNNP model and fitting it to itself using V_m_ and R_m_. A random distribution was selected starting from the control values of the parameters in the published model and applying the lower and upper limits depending upon the data available from literature from other published human ventricle models [18]–[21], [23], [28]–[30]. R_m_ was calculated at three voltage points: 22.57 mV, 8.084 mV and −59.87 mV. 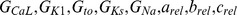 were adjusted. Average and standard deviations of these eight parameters were plotted to check the whether fitting R_m_ is important for model-to-model fit.

After verifying the performance of the algorithm, the TNNP model was fit to APs and R_m_ values generated by a different model of the human ventricular AP, the IMW [23] model. This model exhibits a somewhat different AP morphology from that of the TNNP model, and uses different formulations for some of the ionic currents. This scenario was intended to address the situation where a mathematical model is adjusted to fit experimental data which it may not be able to reproduce exactly. R_m_ was fit at the three voltage points.

### Biological Data Fitting

Two different experimentally recorded APs, each with R_m_ determined at 4 or 5 points during the AP (data sets 1 and 2), were kindly provided by Dr. Kenneth W. Spitzer, University of Utah (unpublished data). This study was approved by the Institutional Animal Care and Use Committee (IACUC) of the University of Utah school of Medicine. The technique used to obtain the experimental data is available in the literature [17]. Briefly, it involved continuous pacing of the cell under current clamp and then at various times during the action potential, switching from current clamp to voltage clamp for ∼30 ms. Instantaneous current-voltage curves were constructed from the V_m_ and I_m_ measured 10 ms following the onset of the clamp. Thus, the experimental protocol is very similar to the one used for simulations as explained earlier in R_m_ evaluation section in methods. Due to the variability in APs, it is not guranteed that voltage is exactly clamped to 10 mV above and below the V_m_ of interest as the voltage while switching to clamp can be slightly different to the voltage recorded from action potential immediately before applying the clamp. An Axoclamp 2B amplifier system was used for current and voltage clamping (switch clamp, 8 kHz chopping frequency). The sampling frequency for all signals was 20 kHz. The pipettes (resistance ∼3 M 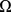) were coated with Sylgard to decrease capacitance. All V_m_ values were corrected for the 10 mV liquid junction potential. The bathing solution contained (mM): 126 NaCl,11 dextrose,4.4 KCl, 1.0 MgCl_2_, 2.2 CaCl_2_, 24 Hepes, 12.9 NaOH (pH7.4). A amphotericin perforated patch was used for data set 1. The pipette solution for data set 1 contained: 123 mM K glutamate, 10 mM NaCl, 10 mM KCl, 10 mM Hepes(free acid) titrated to pH 7.2 with 8 mM KOH, amphotericin ∼250 µg/ml. The temperature of the bathing solution was 36°C. The pacing cycle length was 1 sec. A ruptured patch was used for data set 2. The pipette contained (mM): 123 potassium glutamate, 10 NaCl, 5.5 dextrose, 5 dipotassium ATP, 1MgCl_2_, 10 Hepes, 12 KOH (pH 7.1). The temperature of the bathing solution was 34°C. The pacing cycle length was 2 sec.The current was measured 10 ms after the voltage clamp.

Since these experimental data were obtained from rabbit ventricular cells, we opted to use the UCLA rabbit model [31]. The eight major conductances 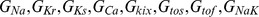 were varied to fit the experimental data. R_m_ was calculated at five voltage points (24 mV,−1 mV,−23 mV, −28 mV, and −81 mV) for data set 1 and four voltage points (32 mV, 28 mV,−21 mV and −78 mV) for data set 2. These values were chosen as they correspond to the experimentally obtained data.

## Results

### R_m_ calculation

To illustrate the ambiguity in parameter values when R_m_ is not considered, single cell simulations were run for the TNNP model using two different sets of parameters (Table 3). Fig. 2A show the APs for parameter set 1 and set 2. It can be seen that two drastically different combinations of ionic conductances resulted in nearly identical APs in the single cell, as previously demonstrated [27]. Fig. 2B shows the tissue APs for the two parameter sets. Note that the APs are quite different in the tissue setting, despite being nearly identical in the single cell simulations. APD_90_ for parameter set 1 for tissue has been increased by approximately 5.95% from the control value of APD_90_ of single cell simulation whereas for parameter set 2 it has been reduced by 45.23%.

To see whether R_m_ was different in single cell with these two sets of parameters, R_m_ was evaluated in the single cell model at nine different points (shown by star markers in Fig. 2A). R_m_ changed substantially during the time course of the AP, and, moreover, R_m_ curves for the two parameter sets were very different. As shown in Fig. 2C, R_m_ for parameter set 1 (red curve) is approximately ten-fold higher during the repolarization phase as compared to parameter set 2 (blue curve). This demonstrates that R_m_ contains information independent of AP shape. In tissue, the cells are connected by gap junctions that are responsible for charge transfer between cardiomyocytes [28]. R_m_ provides information about how sensitive the AP waveform is to current flow among adjacent cardiomyocytes. In the single cell, a large efflux cancelling a large influx, or a small efflux cancelling a small influx, may yield the same net current and, thus, the same AP. However, these two scenarios will likely have different R_m_ and could be distinguished by taking R_m_ into account.

### Model Self Fitting

**Figure 3:**
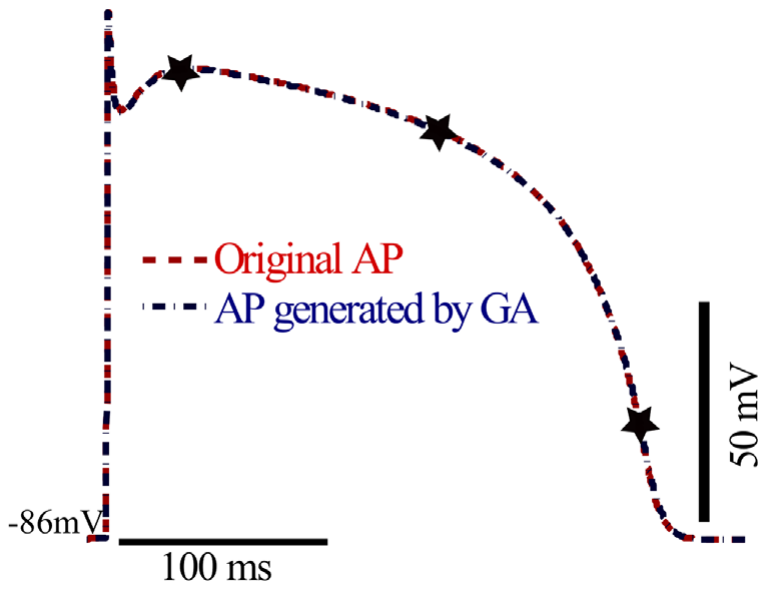
Fitting TNNP model to itself. The red dashed curve shows the single cell AP for TNNP model as published [22]. the blue dashed-dotted curve shows the single cell AP waveform generated by genetic algorithm after fitiing AP+R_m_. Membrane resistance was evaluated and fitted at three different voltage points as indicated by the markers.

The performance of the algorithm as described in the Methods section was first verified by by successfully fitting the TNNP model to itself, as shown in Fig 3. The GA fit was not improved by adding R_m_, and, hence, the curve for AP fit overlies exactly the AP+R_m_ fit. The fact that the AP+R_m_ fit did not perform better is expected, as this was a model fit to itself for which a perfect solution exists, making this fitting problem relatively straightforward. Even using only 3 resistance points, an excellent fit was achieved in only 16 iterations. The fit AP was indistinguishable from the target AP. Using only the AP, the fit took 50 iterations. The value of MSE for the action potential of the TNNP published model and the AP generated by GA using AP+R_m_ fit was 0.1 mV.

### Model to Model Fitting

**Figure 4:**
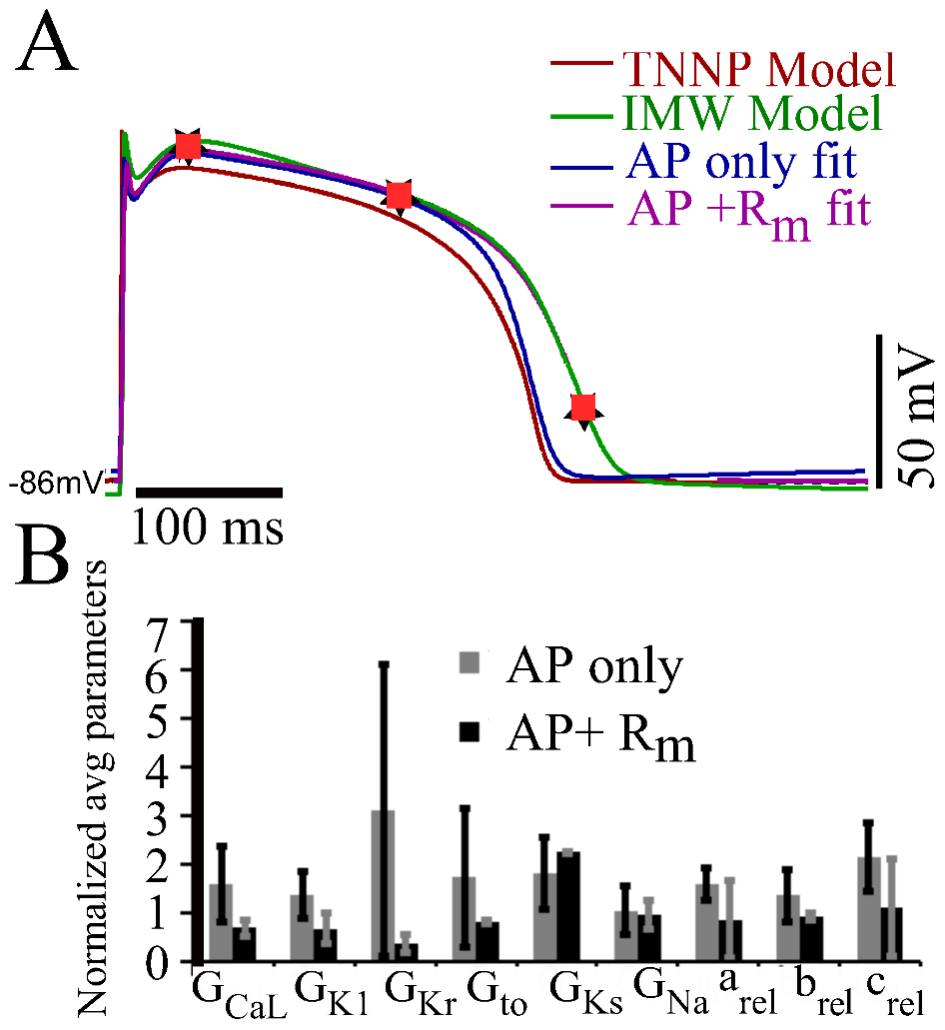
Model-to-model fit: TNNP ventricle model fitted to the IMW ventricular model. (A): The TNNP (red) was fit to the IMW model (green), using only the AP (blue), or the AP+R_m_ (magenta) measured at three different points (black star markers). Orange square markers represent the corresponding voltage points in AP+R_m_ fit curve obtained from genetic algorithm (B): Plot of normalized average and SD of parameters from the pareto set generated from the genetic algorithm output from AP only (gray) and AP+R_m_ fits (black).

We attempted to fit the TNNP model to the IMW model, using only AP morphology and using both AP+R_m_. Results are shown in Fig. 4A.

The AP+R_m_ fit matches the target APD of IMW model very well as compared to AP only fit. There was a depression of the action potential amplitude by 7.9% and 8.8% for AP only and AP+R_m_ fits respectively. The difference in APD_90_ of published IMW model and the APD_90_ of AP generated by AP only fit is 13.04% wheras APD_90_ difference of actual IMW published model and AP generated by AP+R_m_ fit is 2.79%. MSE voltage and MSE R_m_ by decreased by 19 fold and 29 fold respectively by fitting AP and R_m_ simultaneously as compared to fitting AP only. By adding another constraint, R_m_, that has functional relevance, we are providing more information to the GA, and, hence, narrowing down the problem. R_m_ is a relevant parameter as many different conductance values can give similar APs. A large outward current cancelling a large inward current is indistinguishable from a small inward current cancelling a small outward current. Adding R_m_ at few voltages to the AP fit led to an improvement in fitting.

Fig. 4 B compares the average and standard deviation of the normalized parameter adjustments obtained for the two different fitting protocols over 100 and 18 fits for AP only and AP+R_m_ fit respectively. The average and standard deviation for each parameter was normalized by the control value of the parameter in the TNNP published model. The variability in almost every estimated parameter values was greatly reduced by considering R_m_. For instance, variation in 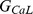 decreased by 80.5% and for 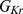 was reduced by 93%. The variability for the conductances was reduced from 40.1% (in 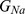) to 95.7% (in case of 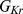).

### Tissue Simulations

**Figure 5:**
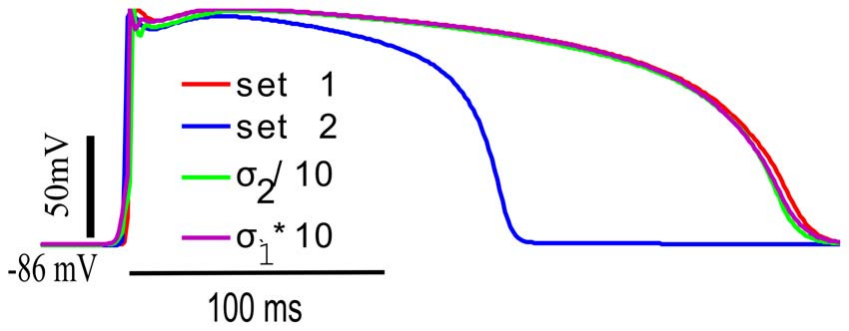
Effect of changing tissue coupling with the two parameters sets of TNNP model. Red and blue curve show the APs for parameter set 1 and 2 for TNNP model. The green curve shows the AP for parameter set 2 when the coupling has been decreased by a factor of 10. The purple dotted curve shows the curve for parameter set1 with the coupling increased by a factor of 10.

We also tried tissue simulations as shown in Fig. 5 to determine the effect of coupling resistance on the AP in tissue, and how it relates to membrance resistance, R_m_. Red and blue curves in Fig. 5 show the AP for parameter set 1 and set 2 respectively. In the early phase of AP near plateau R_m_ for parameter set 2 R_m_ at this point decides what is going to happen in the later phase of the AP. For parameter set 2, interconnecting cells in tissue with normal intercellular coupling (the blue curve) results in shortened APD. Decreasing the longitudinal and transverse intracellular conductivities of parameter set 2 by a factor of 10 resulted in action potetnials in the tissue simulations that were similar to those in a single cell, i.e., the APD increased as the cells were decoupled. Conversely, increasing tissue coupling with parameter set 1, which has lower R_m_ at first four points is already well coupled.

### Fitting Biological Data

**Figure 6:**
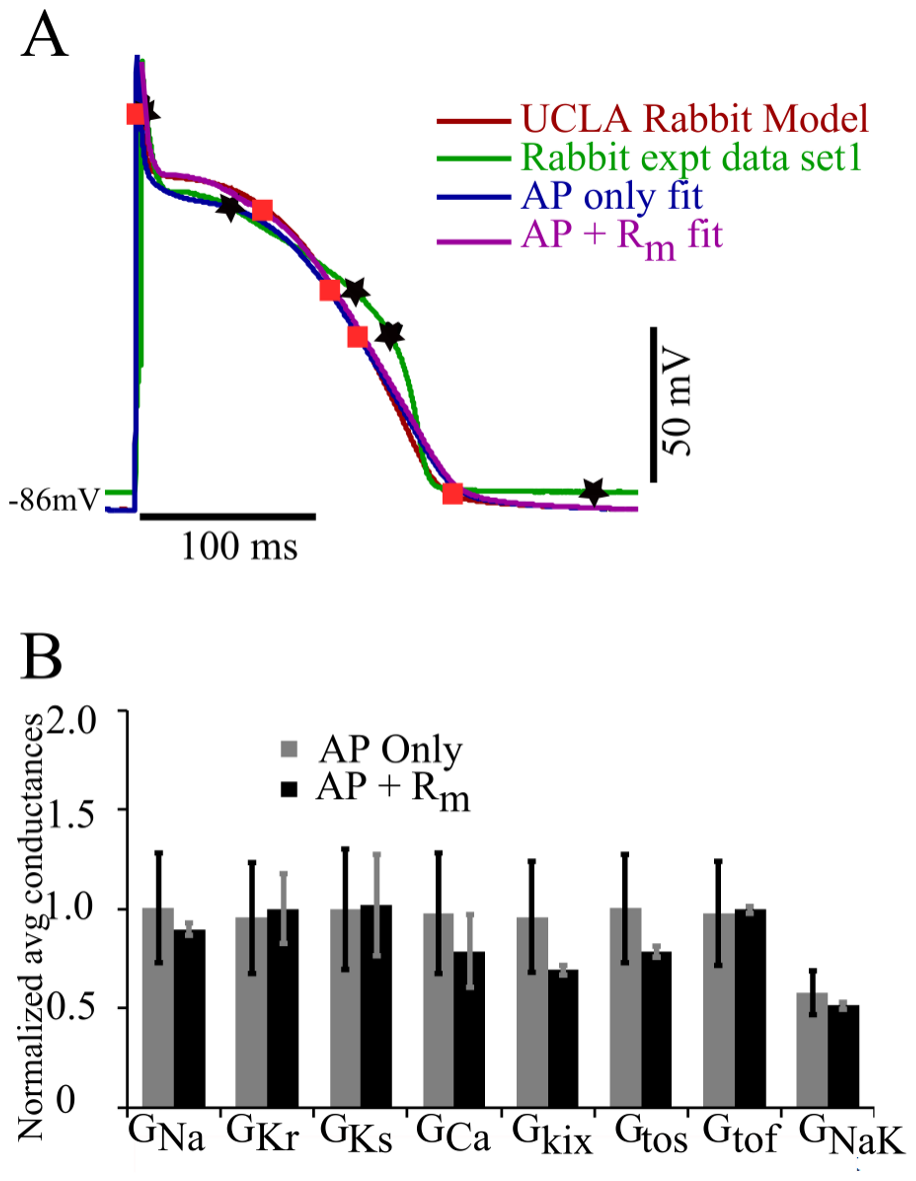
Fit of UCLA rabbit ionic model to experimentally recorded AP data set 1. (A) The UCLA rabbit ionic model (red) was fit to experimental data set 2 (green) using just the AP (blue), and the AP+R_m_ (magenta) measured at 5 different points (black star markers). Orange square markers represent the corresponding voltage points in AP+R_m_ fit curve obtained from genetic algorithm. (B): Plot of normalized average and SD of parameters from the pareto set generated from the GA output from AP only and AP+R_m_ fits. Bars show normalized averages with SD for the optimal values of particular parameters with AP only fit (grey) and AP+R_m_ fit (black).

**Figure 7:**
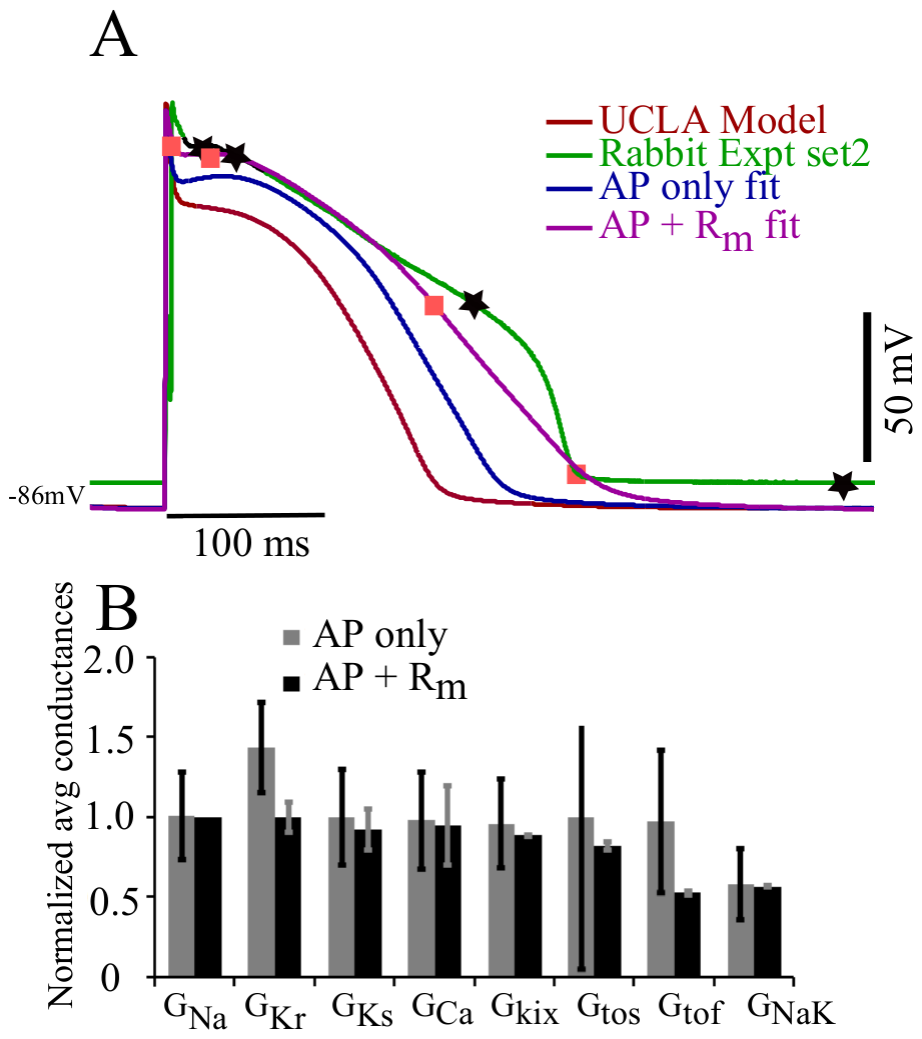
Fit of UCLA rabbit model to experimentally recorded AP data set 2. (A): The base UCLA rabbit ionic model (red) was fit to experimental data set 2 (green) using just the AP (blue), and the AP+R_m_ (magenta) measured at 4 different points (black stars). Orange square markers represent the corresponding voltage points in AP+R_m_ fit curve obtained from genetic algorithm. (B): Plot of normalized average and SD of parameters from the Pareto set generated from the genetic algorithm output from AP only and AP+R_m_ fits. Bars show normalized averages with SD for the optimal values of particular parameters with AP (grey) and AP+R_m_ fits (black).

For further testing the versatility of the approach, we also fit to experimental R_m_ and AP data. Fig. 6A shows the fitting results for first data set and where R_m_ was calculated. Absolute values of R_m_ at the five points were 50.0, 330.0, 1896.0, 123.0, and 9.0 M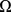 respectively. We again attempted to determine the best-fit model parameters by fitting with and without R_m_. MSE voltage decreased by 4.2% and MSE of R_m_ decreased by 11.42% by using R_m_ Fig. 6B compares normalized averages and SDs of the optimal parameter values obtained from the AP only fit to those obtained from the AP+R_m_ fit. There was not much difference in the average parameter values obtained from the two methods. However, the variability in the parameter values obtained was greatly reduced, by more than a factor of 5, for 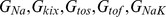 when objective functions for R_m_ were included in the algorithm. The variability in the other parameters were more modestly reduced, in the 10–30% range. We repeated the same procedure for a second experimental data set for which the results are shown in Fig. 7A. The absolute R_m_ values were 23.5, 30.5, 390.0, and 16.7 M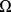 at the four markers shown in Fig. 7A at voltage points 32 mV, 28 mV,−21 mV and −78 mV. The AP for the experimental data was entirely different for data sets 1 and 2 as cells and the pacing cycle length were different. While neither fitting protocol of genetic algorithm yielded a very close fit to the AP shape, the AP+R_m_ fit was better than the AP only fit. Given our mathematical formulation and constraint of parameters, no parameter set could produce a good match to the experimental data set. Moreover, the problem was more challenging in this case since the experimental AP curve was vastly different from TNNP published model. Absolute error difference between APD_90_ for AP+R_m_ fit and actual experimental data was 2.87% whereas in case of AP only fit it was 18.9%. Furthermore, the difference in peak-overshoot-potential for AP+R_m_ fit from experimental data was 0.84% whereas in case of AP only fit this difference was 9.1%. Thus, AP+R_m_ fit decreased the variations in APD and POP. Furthermore, while average parameter values are mostly similar between the two protocols, the variability in parameter values obtained was again reduced for the AP+R_m_ protocol. This was particularly evident for the 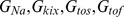, and 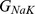 parameters, for which variability was reduced by at least 34-fold. Variability in parameters 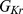 and 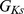 was reduced by 3 and 2-fold, respectively, while in the case of 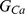 the variability was reduced by 18.6%.

**Figure 8:**
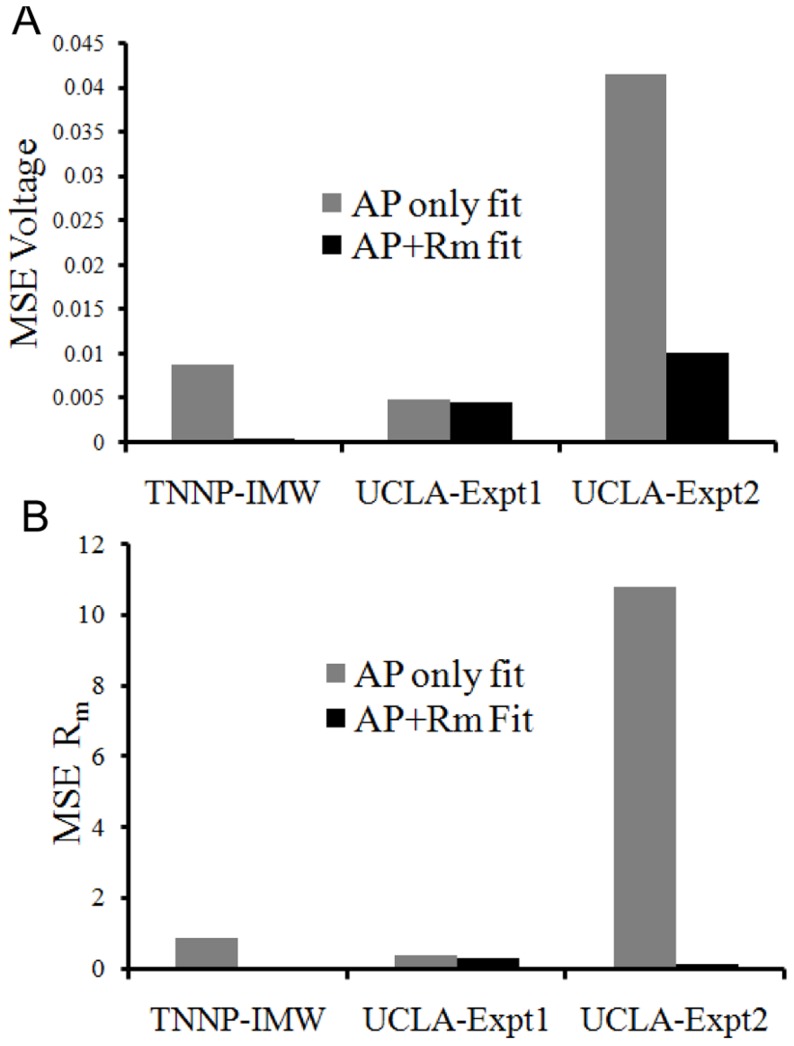
Mean square errors. Error for fitting the TNNP to the IMW model (TNNP-IMW), and fitting the UCLA rabbit model to experimental data sets 1 (UCLA-Expt1) and 2 (UCLA-Expt2) are shown. Grey bars shows MSE for fitting AP only while black bars show the MSE of fitting AP+R_m_.(A). MSE of AP voltage normalized by square of height of AP. (B) MSE of R_m_ normalized by square of actual membrane resistance at particular voltage point.

The mean square error for the cases, fitting TNNP to IMW model fitting, and fitting UCLA model to experimental data set 1 and 2, were measured (Fig. 8). The error was considerably less in the case of AP+R_m_ fit in TNNP to IMW fit and UCLA to experimental data set 2 as compared to AP only fit. MSE voltage for experimental data set 2 has been decreased 4 fold whereas MSE of R_m_ is decreased by 68 folds by fitting AP and R_m_ simultaneously as compared to fitting AP only A possible reason for the small difference for fitting experimental data set 1 was its similarity to the UCLA rabbit model. Thus, the AP only fit was sufficient to fit the model to the data. Adding R_m_ did not have much scope to improve the results to a great extent. Whereas in the second set of experimental data, the AP shape was entirely different from the published TNNP ventricle model and thus there was a strong need to fit R_m_ as well along with fitting AP.

There was a seven fold overall increase in the computational cost of an iteration for fitting additional parameter R_m_ at few points. There was a decrease in the number of iterations from 100 in the case of AP only fit to 18 for the AP+R_m_ fit, but still 18 iterations took 25% more time than 100 iterations of AP only fit.

## Discussion

This study puts forth a method for enhancing fitting of APs in single cell models. We propose adding R_m_ as an objective, beyond just AP morphology, and demonstrate that it has several benefits: It leads to less variability in the parameters values obtained, reduces computation, as well as leads to better tissue level behaviour.

### R_m_ measurements

Take the current flow of ion *X* through a channel represented by a Hodgkin-Huxley formulation, 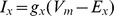, and, consequently 
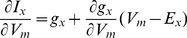
(7)


It can be seen that the channel conductance is composed of two terms, the first being the chord conductance (*g_x_*), and the second being a function of the driving force and the rate of change of the chord conductance, and can be negative. Which term dominates will depend on many factors which change throughout AP. Pumps and exchangers also have nonlinear conductances. The total cell conductance is, then, the summation of a set of nonlinear conductances which makes it noninutuitive.

We chose different voltages at which to measure R_m_ instead of times post-activation. This is because currents have a strong voltage dependence so by sampling at different voltages, we are sure to get different distributions of conducting ionic channels. When a particular channel is more active during a certain phase and contributes a large portion of the membrane conductance, this provides more information to the GA to help fit its absolute magnitude.

If we simply chose times at which to compute R_m_, we could get multiple samples from the plateau which would be very similar. If the two cells had very different APs, we would end up sampling very different states and R_m_ comparisons would not be meaningful. Finally, we would have had to adjust the algorithm to the particular APD to ensure proper sampling of all states.

We chose a 5 ms delay after the voltage clamp onset to allow very fast transients to die down, and the major currents to stabilize. Sodium, calcium, and transient outward channel activation gates have kinetics on the order of 1–5 ms [22]. Within this short time period (5 ms) as compared to the duration of an AP, activation of major currents such as I_Na_, I_CaL_, I_to_ and inactivation of I_Kr_ etc. are stabilized and all the slower kinetics are assumed to be frozen. This definition of membrane resistance has the advantage that it can be experimentally assessed, and is relevant to dynamics on the timescale of an AP. Table 4 shows the computed values of R_m_ after 5 and 10 ms after the start of clamp pulse to see the difference between the R_m_ values computed. Measuring R_m_ 5 or 10 ms after the onset of clamp pulse does not make a substantial difference except at the starting two points during the plateau phase of AP. Except for these points, on average, there was only 1.1% difference in R_m_ measured. If we include the first two points, it gave an average percent difference of 4.6%. We verified our choice of timing by measuring the MSE in APs fit with R_m_ measured 5 ms and 10 ms after the clamp pulse. It was observed that the difference in MSE voltage and MSE R_m_ for TNNP to IMW model fit was only 0.03% and 0.04% respectively. Furthermore, for the theoretical R_m_ calculation, capacitive transients do not need to be dealt with, so 5 ms is acceptable. We conclude that differences between using 5 ms and 10 ms delay to measure R_m_ are negligible in terms of the quality of the resulting fit. For experimental R_m_ measurements, a 10 ms delay was chosen due to the presence of capacitive transients. If we waited longer, the state would have evolved too much and the response would have been more a function of the clamp voltage.

**Table 4:** R_m_ measured 5 ms and 10 ms after start of clamp pulse.

	Time (ms)	Voltage (mV)	R_m_ after 5 ms	R_m_ after 10 ms	Error
	3020	14.5	5.6	6.8	20.9%
	3035	19.9	7.0	7.9	12.8%
	3050	22.4	7.4	7.6	2.2%
	3080	20.8	8.4	8.5	1.4%
	3140	14.4	13.8	14.0	1.6%
	3170	9.7	46.8	47.0	0.5%
	3260	−30.4	149.3	152.1	1.9%
	3276	52.4	4.2	4.2	0.0%
	3320	−86.0	0.492	0.492	0.0%
**% Error for all points**					4.6%
**% Error except first two points**					1.1%

### Justification of Fitting R_m_

The results shown in Fig 2 revealed that different sets of parameters produce almost similar AP in single cell but entirely different morphology in the tissue, highlighting that models producing good AP fits in single cell simulations may sometimes fail to reproduce the AP in tissue simulations. For example, recent detailed atrial [24] and ventricular [18] ionic models have failed to propagate in tissue, despite faithfully reproducing expected isolated behavior. In tissue, cells are interconnected through gap junctions and interact electrically with neighbouring cells. If we take the example of two cells connected to each other, cell 1 (source) is more depolarized than cell 2, so it will try to depolarize cell 2 whereas the sink has opposite effect on the source (repolarizing influence). The membrane resistance, R_m_, relates the change in membrane voltage to the current by these source-sink interactions. If R_m_ is high, a small current produces a large change in voltage. If R_m_ is low, a large current produces a small change in voltage. R_m_ curves for two model parameter sets were very different (Fig. 2B).

For parameter set 1, R_m_ was approximately ten-fold higher during repolarization as compared to set 2. R_m_ is maximum during depolarization as many channels are voltage activated and close. The shorter the APD, the lower the average R_m_ must be. This is because the amount of charge to dissipate through the membrane is a function of the peak voltage. The average current flow will be this charge divided by the APD. R_m_ must, therefore, be lower with the higher current to keep the voltage at the same level as with the longer APD.

R_m_ relates the change in membrane voltage to an injected current (or in the case of cells in tissue, current flowing through gap junctions to/from adjacent cells). If R_m_ is high, a small current produces a large change in voltage. If R_m_ is low, a large current produces a small change in voltage. In tissue, the current through gap junctions depends on source-sink interactions and thus coupling between the cells. Thus, membrane resistance interacts with coupling resistance in determining the behaviour of cellular APs in tissue, and properly fitting R_m_ yields better results in tissue simulations.

This further emphasizes the importance of R_m_. If considering only net current flow while fitting maximal conductance values, there could be a large influx cancelling large efflux due to ionic currents with large chord conductances, or a small efflux cancelling small influx due to ionic currents with low chord conductances, in both cases resulting in a similar net current. One may argue that the net flux of a particular ionic species will have other effects beyond direct membrane voltage changes and that these effects should also direct the fitting. However, given the complex feedback interactions and different sensitivities to model parameters, more direct fitting data seems to be of great help. Thus, R_m_ is a vital parameter that can provide information regarding ionic currents that is not sufficiently provided by just the shape of AP. Hence, we propose that fitting net membrane current and R_m_ at different voltage points in the AP cycle can overcome this problem.

### Computation Time

Including R_m_ improves the fit and reduces number of runs approximately five fold at the price of more expensive iterations. Each R_m_ measurement requires two runs, one to increase and one to decrease V_m_. This aspect of the computation was not fully optimized in this implementation. For example, for every R_m_ measurement, we reran the initial 3 seconds. We could have saved the state immediately before the R_m_ calculation, performed the calculations, and then continued from the saved state. Thus, although we observed a sevenfold increase in objective function evaluation time, this could be reduced significantly, theoretically to 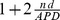 where *n* is the number of R_m_ points and *d* is the duration of the voltage clamp, notwithstanding system time to launch any additional jobs required. In any case, the single cell simulations take on the order of seconds and GAs parallelize well, so computation time is not an obstacle and is actually significantly reduced.

### Effects of fitting with R_m_

The TNNP human ventricle model fit to the IMW model both with and without incorporating R_m_ data. Parameter values obtained with both methods were similar on average after 100 runs. However, the variability in 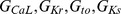 and 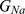 was considerably smaller for combined AP and R_m_ fit compared to AP only fit, and also brings AP closer to the desired AP curve of IMW model. Hence, addition of one extra objective, (R_m_, at a few voltages) to the AP fit, improved the fit to the desired AP curve while reducing variability in the solutions obtained. This also reduced the number of runs to perform from 100 to 18. The mean square error was reduced 19-fold by fitting R_m_ at three points during the AP.

The UCLA rabbit model was fit to the experimental rabbit ventricular data sets 1 and 2, with R_m_ determined at 5 and 4 points respectively during the AP. For data set 1, the AP of the UCLA model is quite similar to rabbit experimental data set 1. There was not much scope for fitting by the additional parameter R_m_ (magenta curve) at five voltage points as compared to fit AP only (blue curve). Thus there was not much change in the shape of AP while fitting AP only or AP+R_m_. Fig. 4B shows that there is not a significant change in the average for both AP only and AP+R_m_ fit, but the variation for parameters 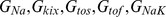 was reduced considerably for the AP+R_m_ fit along with a reduced number of runs, from 100 to 18. The experimental behaviour was not exactly replicated during repolarization and also for 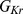 and 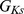 conductances, variation was not significantly decreased as compared to the model-to-model fit. The possible interpretation of this can be that these are the currents responsible for this part of the AP, and the problem is more challenging to fit than the model-to-model fit. There might not be a best possible solution to fit this part of AP as well without making other parameters worse. The MSE was only reduced by a factor of 1.1 by fitting R_m_. So, since the UCLA model AP and experimental data set 1 AP curve are not very different, the AP only fit was sufficient for reproducing the experimental results.

The experimental data set 2 for AP and R_m_ at 4 voltage points in Fig. 7A is quite different from UCLA rabbit model AP+R_m_ fit together is closer to the experimental AP than the AP only fit (blue curve of Fig. 7A). The variation in parameters 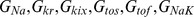 was reduced further along with a reduced number of runs to perform from 100 with AP only to 14 for AP+R_m_ fit. Hence, addition of one extra objective, (R_m_, at a few voltages) to the AP fit, improves the fit to the desired AP curve while reducing variability in the solutions obtained. It also reduced MSE 4 fold. It has been noticed that experimental data. The possible reason can be that the 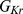 and 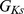 which are the dominating currents during this phase of AP did not vary much in average and variation from the AP only fit. For cases where the target AP is very different from the model, R_m_ improves the fitting.
